# Chemical, Aroma and Pro-Health Characteristics of Kaffir Lime Juice—The Approach Using Optimized HS-SPME-GC-TOFMS, MP-OES, 3D-FL and Physiochemical Analysis

**DOI:** 10.3390/ijms241512410

**Published:** 2023-08-03

**Authors:** Martyna Lubinska-Szczygeł, Żaneta Polkowska, Małgorzata Rutkowska, Shela Gorinstein

**Affiliations:** 1 Department of Analytical Chemistry, Faculty of Chemistry, Gdańsk University of Technology, 80-233 Gdansk, Poland; malgorzata.rutkowska@pg.edu.pl; 2 Institute for Drug Research, School of Pharmacy, Hadassah Medical School, The Hebrew University, Jerusalem 91120, Israel; shela.gorin@mail.huji.ac.il

**Keywords:** kaffir lime, gas chromatography, plasma-optic emission spectrometry, 3D fluorometry, optimization, validation, aroma properties, health benefits

## Abstract

The study aimed to provide the chemical, aroma and prohealth characteristics of the kaffir lime juice. A procedure using solid-phase microextraction with gas chromatography (SPME-GC-TOFMS) was optimized and validated for the determination of terpenes of kaffir lime. Main physicochemical parameters: pH, vitamin C, citric acid and °Brix were evaluated. Micro- and macro elements were determined using microwave plasma optic emission spectrometry (MP-OES). The binding of kaffir lime terpenes to human serum albumin (HSA) was investigated by fluorescence spectroscopy (3D-FL). β-Pinene and Limonene were selected as the most abundant terpenes with the concentration of 1225 ± 35 and 545 ± 16 µg/g, respectively. The values of citric acid, vitamin C, °Brix and pH were 74.74 ± 0.50 g/kg, 22.31 ± 0.53 mg/100 mL, 10.35 ± 0.70 and 2.406 ± 0.086 for, respectively. Iron, with a concentration of 16.578 ± 0.029 mg/kg, was the most abundant microelement. Among the macroelements, potassium (8121 ± 52 mg/kg) was dominant. Kaffir lime binding to HSA was higher than β-Pinene, which may indicate the therapeutic effect of the juice. Kaffir lime juice is a source of terpenes with good aromatic and bioactive properties. Fluorescence measurements confirmed its therapeutic effect. Kaffir lime juice is also a good source of citric acid with potential industrial application. The high content of minerals compared to other citruses increases its prohealth value.

## 1. Introduction

Kaffir lime is one of the most popular fruits in Southeast Asia. Unlike many citrus fruits, the main part of the fruit consumed is not the juice but the aromatic leaves. The juice is not consumed directly because of the extremely tart and often bitter taste [1,2]. In traditional medicine in some Asian countries, juice is often used in shampoo because of its antidandruff properties or as a cleanser for clothing [3]. Detailed chemical characteristics of the fruit make it possible to detect specific compounds responsible for the health-promoting effect of the fruit. In the case of citrus, their bioactive effect is due, among others, to the presence of terpenes [4]. Terpenes are secondary metabolites of many plants, produced to fulfil specific biological and ecochemical functions, such as hormone biosynthesis, but also protection against UV radiation and photo-oxidative stress, but also as pest and toxin repellants, growth regulators, pollinator attractors, photosynthetic pigments and electron acceptors [5]. Less volatile, bitter or toxic terpenes are produced by plants as protection against microbes and insects [6]. Terpenes are used primarily as fragrances in new perfumes and as additives to creams, lotions, or shampoos. In addition, some functionalized terpenes also show bioactivity against certain types of cancerous, bacterial, or viral cells. Therefore the interest in such compounds is constantly growing [7]. It is recommended that the terpenes used in the food, cosmetic and pharmaceutical industries should be of natural origin.

Determination of chemical, aroma and prohealth characteristics of fruits is extremely important from the point of view of their use in various industries. Since terpenes are the main group of chemicals in citrus, much attention is paid to them. Considering the variable factors that may affect the result of the determination of terpenes in fruit juice samples, such as growing conditions, degree of maturity or storage conditions, an optimized and validated methodology for the determination of these compounds should be developed, which will ensure repeatability and reliability of results regardless of external factors. This is especially important for kaffir lime juice, which is not directly consumed. It is known, however, that it is used in folk medicine or as a home cosmetic, but its detailed health-promoting properties have not been fully investigated. Kaffir lime is an underrated fruit and a good source of many chemical compounds, especially since the juice is not consumed due to its tart taste. To use a given raw material in the industry, methodologies for its analysis must be developed. In previous literature reports, terpenes turned out to be the main group of chemical compounds in kaffir lime. It was decided to focus on this group and optimize and validate the analytical methodology for it.

The study of the volatile fraction of citrus fruit is a popular element of food research [8]. However, many of the performed approaches concern the qualitative or semiquantitative analysis of volatile terpenes under non-optimized conditions. For quantitative analyzes, a common approach is to run an untargeted analysis to determine the overall fragrance profile. In the case of kaffir lime, previous untargeted studies have shown that the aroma profile of the juice is primarily terpenes such as ƴ-Terpinene and Terpinen-4-ol [9]. However, the research was conducted under non-optimized conditions. In line with recent trends, solvent-free extraction techniques such as solid phase microextraction (SPME) should be used in volatile fraction studies. The efficiency of HS-SPME of monoterpenes can be affected by several factors, including the mass of the sample and the addition of salt, which increases the ionic strength, extraction time and temperature. Henceforth, an optimized analytical method for targeted terpenes determination is crucial to facilitate the characterization.

The first objective of the study was the development of an analytical procedure based on the HS-SPME in combination with gas chromatography with time-of-flight mass spectrometry (GC-TOFMS) for targeted analysis of terpenes in kaffir lime juice sample. Optimization of major HS-SPME conditions using fractional factorial design (FFD) was performed. The second objective was the validation of the elaborated procedure and application to real samples. Moreover, a characteristic of kaffir lime, including sugar, citric acid and micro and macroelements, was performed. Bioactive compounds’ interaction with albumins significantly affects their transport and biological metabolism [10]. To better understand terpenes’ prohealth activity, their binding properties to human serum albumin were measured using three-dimensional fluorescence analysis (3D-FL). The results provide a background for using kaffir lime juice as a functional food or additive cosmetic and pharmaceutical industry. The conducted research complements the previous research on the antioxidant properties of kaffir lime juice and its therapeutic effect on the human body [9,11]. The last goal was to provide the fruit characteristics of major by-products, which can be obtained from kaffir lime or used in citrus waste management processes. For this purpose, a novel analytical method using microwave plasma optic emission spectrometry (MP-OES) was provided. To our knowledge, lime juice research has not been exhausted yet. The paper provides new information about the different branches of science and the different kinds of industry.

## 2. Results and Discussion

### 2.1. Terpenes’ Content

#### 2.1.1. SPME Optimization

Fractional factorial design is a screening method that assesses the effect of certain factors in minimum runs. It is relatively advantageous compared to the full factorial design as it requires many experiments [12]. The design matrix and the corresponding response of fractional factorial design (FFD) experiments were used to determine the influence of the four independent variables, including the mass of sample (X_1_), mass of added salt (X_2_), extraction time (X_3_), extraction temperature (X_4_) for the extraction and enrichment of main terpenes of kaffir lime juice. Based on the experimental design generated by FFD, 11 extraction processes were performed. The response for each run in the experimental Fractional Factorial Design was expressed as the value relative to the maximum yield obtained (%) for each level of main terpenes in kaffir lime juice (Table 1).

The model’s efficiency was evaluated using analysis of variance (ANOVA). Table 2 presents the results of the regression model for independent variables and their interactions. Based on the results, it can be concluded that all of the variables (X_1_, X_2_, X_3_, X_4_) are statistically significant (*p* < 0.05) in relation to their influence on the isolation and enrichment of main terpenes of kaffir lime juice. Moreover, the 2-way interaction between variables X_1_ (mass of the sample) and X_4_ (extraction time) is also significant. High and very close values of regression coefficients (R^2^ = 99.82%, adjusted R^2^ = 99.12%) evidenced a good correlation between measured and predicted data.

To check the adequacy of the experiment of each of the main terpenes in kaffir lime juice, a model for each compound was constructed, and the coefficient of determination R^2^ was considered (Table 3). The model’s coefficients were confirmed to provide a high correlation between the experimental and predicted values for each terpene.

**Figure 1:**
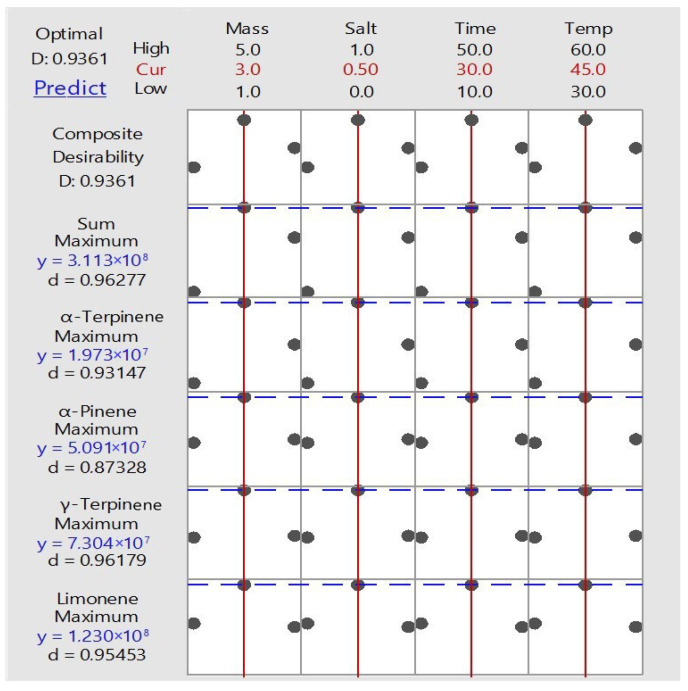
Results of Multiresponse Prediction of the selecting of optimal parameters of extraction of terpenes from kaffir lime juice.

To select the optimal conditions of isolation and enrichment of the main terpenes of kaffir lime juice, Multiresponse Prediction (MRP) was performed (Figure 1). MRP is a method for identifying the combination of input variable settings that jointly optimize a set of responses. *X*-axis shows the optimum values of the variables for a desired response. Y-axis expresses various responses and targets achieved by performing experiments with composite desirability (D) and individual desirability (d) values. The vertical red lines on the graph represent the current settings. The horizontal blue lines represent the current response values.

The proposed ordinates and optimal conditions for HS–SPME by MRP are shown in Table 4.

#### 2.1.2. Method Validation of HS–SPME GC–TOFMS

The validation parameters are presented in Table 5. The determination coefficient (R^2^) ranged from 0.9915–0.9995. The method’s precision was evaluated by assessing repeatability (intra-day) and intermediate precision (extra-day). A measure of repeatability, intermediate precision, and reproducibility were the standard deviation and relative standard deviation values. The precision was calculated as the coefficient of variation for all the results obtained in all the analyzed samples using the developed method (expressed as % CV, *n* = 7). CV ranged from 0.22% (α-Pinene) to 9.17% (α-Terpinene). The coefficient of variation of main components does not exceed 3%, and for all the rest determined compounds, CV is lower than 10%, confirming good repeatability and precision of the method. It is assumed that the developed method meets the requirements when the value of the coefficient of variation does not exceed 15% [13].

**Figure 2:**
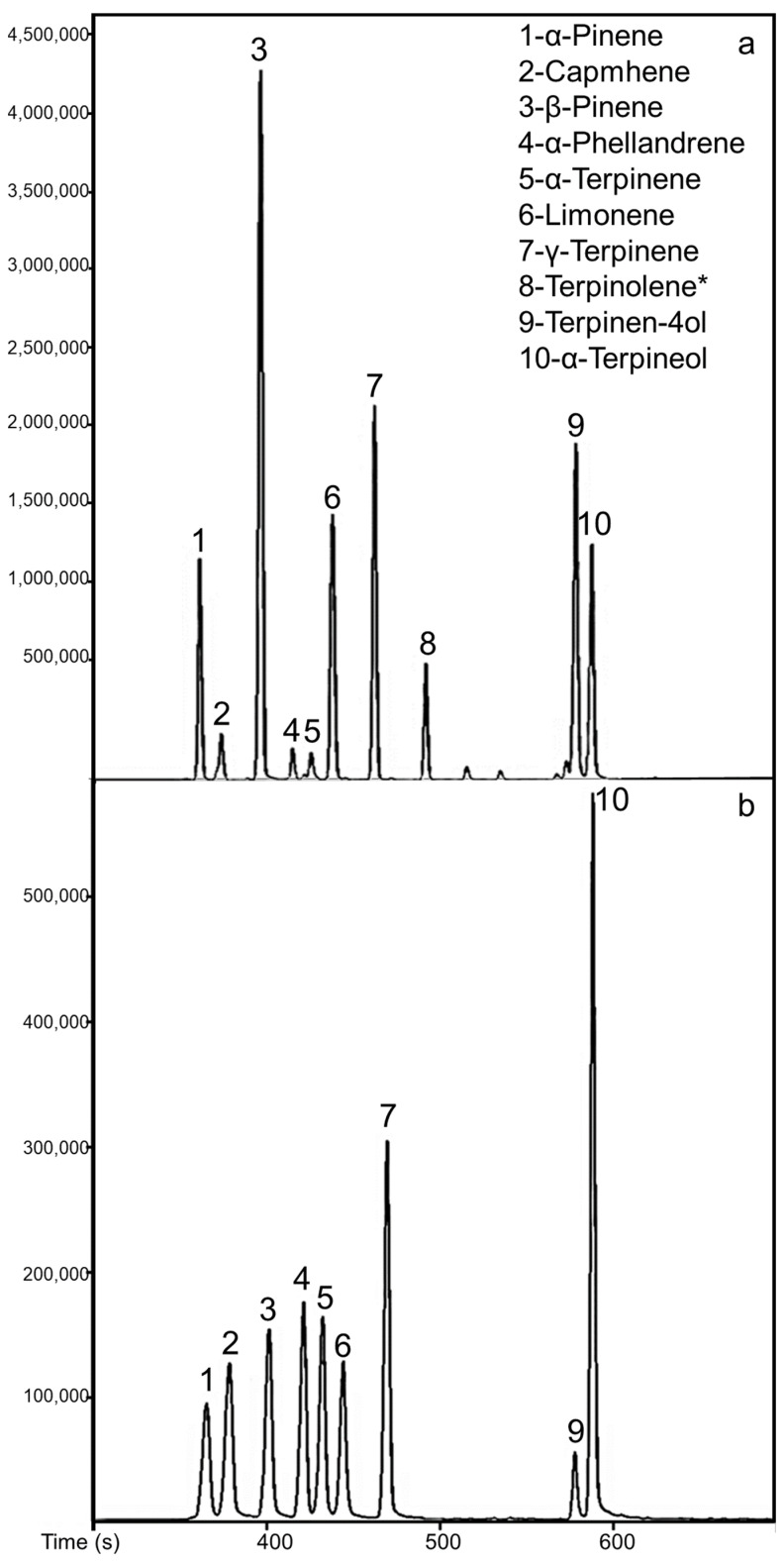
Chromatograms of GC analysis (for the mass 93) of the sample of (**a**) kaffir lime juice, (**b**) terpenes’ standard mixture, performed with the use of optimized extraction conditions, * The compound was investigated in a separate analysis. Therefore it is not shown in the chromatogram.

Certified reference material was not available. Consequently, definitive statements cannot be made concerning accuracy. However, the recovery was calculated according to the results of the analysis of model liquids prepared based on the physicochemical analysis of kaffir lime juice—a model liquid was made corresponding to the juice samples in terms of sugar content (sucrose was the representative sugar), citric acid and vitamin C (which is the main vitamin in citrus fruit juice). The recoveries ranged from 38.33% (Terpinolene) to 127.02% (α-Terpineol). These results show that the developed extraction method applies to assessing studied terpenes. The chromatograms of analysis of the sample and the mixture of terpenes using the optimized method are presented in Figure 2.

#### 2.1.3. Real Samples’ Determination

**Figure 3:**
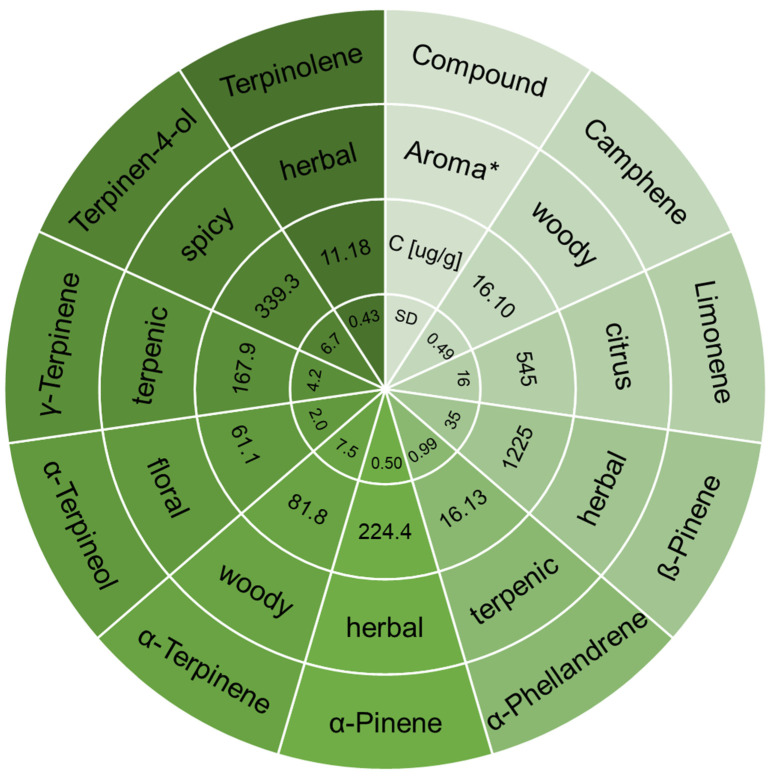
Concentration and aroma properties of main terpenes determined in kaffir lime juice with the use of optimized HS-SPME-GC-TOMFS method, * www.thegoodscentscompany.com (accessed on 14 April 2023).

Using the determined optimal extraction parameters and an appropriately selected temperature program, chromatographic analyzes were performed, based on which calibration curves were prepared that enabled the quantitative determination of selected terpenes in the samples of kaffir lime. The results of the quantitative determination of the main terpenes in kaffir lime juices are presented in Figure 3.

As previously reported, terpenes are the main chemical class of compounds in kaffir lime juice [9]. However, due to factors such as degree of maturity, geographic origin, growing conditions, and harvest time, the terpene profile of the fruit can vary significantly. In pressed juice, time and storage conditions have an additional influence. In the case of terpenes, which are highly reactive and unstable compounds, the influence of temperature, light, enzymes, and the action of microorganisms is not without significance. Terpenes undergo oxidation processes, creating their metabolic pathway and converting to other terpenes, hydroperoxides, monoepoxides, diepoxides and aldehydes [17]. For example, the precursor to α-Terpineol is Limonene and Linalool [18]. According to literature reports, the biotransformation of β-Pinene leads to the formation of Terpinolene [19], from which Terpinen-4-ol is then formed [20]. Terpinen-4-ol can also be synthesized from α-Terpinene [20]. The main terpenes in kaffir lime juice are β-Pinene and Limonene, which are precursors to the synthesis of other terpenes, with concentrations of 1223 ± 35 µg/g and 545 ± 16 µg/g, respectively. In previous reports, Terpinen-4-ol and γ-Terpinene were the most abundant compounds [9]. This indicates greater oxidation of the samples used for previous tests.

Different terpene profiles affect the aromas of the samples but also their bioactive properties. While in previous research, the high content of Terpinen-4-ol could suggest possible anti-inflammatory, antioxidant, antimicrobial and anticancer properties of the juice [21], in the case of high ß-Pinene, it has different prohealth activity. Research provided by Salehi et al. also showed its anticoagulant, antitumor, antimalarial, antioxidant, anti-*Leishmania*, and analgesic effects. The authors summarized its cytogenetic, neuro-, cyto- and gastroprotective, anxiolytic, and anticonvulsant activity [22].

To indicate the intensity of the contribution in creating the aroma of the juice, the odour activity values (OAVs) were calculated by dividing the concentrations of selected terpenes and their odour thresholds (OT) taken from the literature. The OAVs, which is a measure of the importance of a compound to the odour of a sample, were the highest in the case of Limonene and ß-Pinene (Table 6). Comparing the differences in aroma properties with our previous results, the high content of these two terpenes makes the smell of kaffir lime juice more citrus, with herbal accents. Terpinen-4-ol and Terpinolene also contributed to the creation of the aroma with relatively high OAVs. With the comparison with previous research concerning Terpinen-4-ol and γ-Terpinene content, it can be concluded that a greater degree of oxidation, caused by temperature, degree of maturity, or the presence of enzymes and bacteria, may cause changes in the aroma of kaffir lime juice, resulting from the greater presence of Terpinen-4-ol with a woody, and γ-Terpinene with terpenic odour description.

Due to the high content of terpenes, which show numerous bioactive activities [9], kaffir lime juice is a potential candidate for use in the cosmetics industry. It can be used as an additive to hair care products, shampoos, soaps, skin creams, gels, and lotions. This is consistent with literature reports that show that the kaffir lime juice is a natural acid, good for protecting the skin of the head as well as cleaning the residue of soap and shampoo [3]. Due to its pleasant sensory properties will work well as an addition to foot creams, ensuring their pleasant smell. Due to the high content of Limonene, which is known for its ability to fight insects [23], kaffir lime juice will also work as an addition to insect repellents, providing not only effective insect removal but also a pleasant smell. The advantage of this solution will also be the natural origin and non-toxicity of the active substance—terpenes from Kaffir lime juice.

**Table 6:** Aroma intensity properties of kaffir lime juice.

Compound	OT µg/g	OAV	Ref.
Camphene	186	0.086545	[24]
Limonene	0.01	54,488.61	[24]
ß-Pinene	1.5	816.8033	[24]
α-Phellandrene	0.5	27.15541	[25]
α-Pinene	26	8.632425	[26]
α-Terpinene	2.4	34.06655	[27]
α-Terpineol	0.33	185.0893	[28]
γ-Terpinene	1	167.8755	[24]
Terpinen-4-ol	0.34	998.0478	[24]
Terpinolene	0.041	272.705	[24]

### 2.2. Physicochemical Characteristics of Kaffir Lime Juice

The physicochemical characteristics of kaffir lime juice are presented in Table 7. In some countries, citric acid is still produced from citruses as it is economically advantageous [29]. Citric acid is the major organic acid that contributes approximately 90% of the citrus fruit acidity [30]. Among citrus fruits, citric acid is most concentrated in lemons and limes [31]. Previous studies have shown that lemons contain 48.0 g/L of citric acid [31]. The determined kaffir lime’s citric acid content is much higher. Literature researches suggest that kaffir lime can be used as an organic demulsifier formulation and to decrease water hardness due to the high content of citric acid [32,33]. Citric acid extracted from citruses is used as an acidity regulator in beverages, detergents, and for applications other than food, such as cosmetics, pharmaceuticals, and the chemical industry. Using directly consumed lemon juice seems less cost-effective than using Kaffir lime juice, which is food waste [9]. Due to the high content of citric acid, which fights scale deposits in bathrooms, but also due to the presence of terpenes with antibacterial properties and pleasant sensory properties, kaffir lime juice seems to be a very good ingredient in preparations for cleaning bathrooms or toilets.

Brix/acid ratio is considered the best objective measurement that reflects the consumer acceptability of juices [34]. Consumer research proves that the higher the °Brix/acid ratio, the more acceptable the juice is by consumers, which may explain the lack of kaffir lime consumption. To compare, the literature shows that the °Brix/acid ratio of orange juice samples ranges from 15–18 [35].

In the case of the most abundant vitamin in citrus juice—the concentration in kaffir lime juice was determined to be 22.31 ± 0.53. Very similar results were obtained by Najwa et al.—21.58 ± 0.51 mg/100 g [36]. In the same research, orange represented the highest vitamin C content—43.61 ± 1.72 mg/100 g among all citrus fruits. One of the best sources of ascorbic acid, acerola, contains 1500–4500 mg/100 g, which is around 50–100 times more than orange or lemon [37]. Considering the above, Kaffir lime is an average source of vitamin C. However, considering that the juice is waste that can be used to produce dietary supplements or cosmetics, the presence of vitamin C in a relatively large amount is an additional advantage. Vitamin C in cosmetics protects the skin against oxidative stress and fights free radicals.

### 2.3. Micro- and Macroelements Content in Kaffir Lime Juice

**Figure 4:**
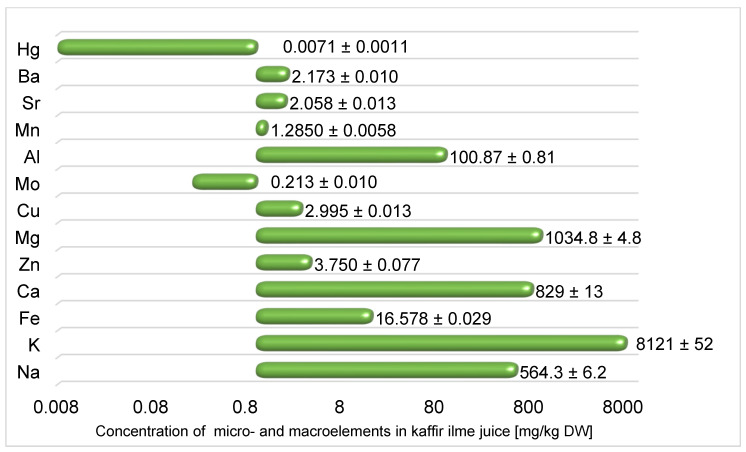
The concentration of micro- and macroelements in kaffir lime juice [mg/kg dry weight (DW)] was determined using the MP-OES technique.

Over the past few decades, several studies on nutritional elements have been conducted to determine their role in the human diet. Heavy metals belong to a group of xenobiotics which are the most commonly controlled harmful components of food or other products due to their ability to accumulate along the food chain. Accordingly, their maximum levels have become global quality standards. Right next to elements considered toxic (e.g., Cd, Pb, Hg), some elements are essential and indispensable in the human diet (e.g., Co, Cr, Fe, Mn, Mo, Ni, Sn, Zn, Ca, Mg and K). However, elevated levels of both essential and non-essential elements can also cause health anomalies. This, therefore, leads to the conclusion that it is so important to determine the level of trace elements in kaffir lime juice [38]. The results of the determination of selected elements in kaffir lime juice are presented in Figure 4.

Macroelements such as magnesium, calcium and potassium in food contribute to the normal functioning of the cardiovascular system, the conduction of nerve impulses and the support of the immune system [39]. As part of the study, of all elements determined, the highest concentrations were found for Mg, Ca, and K. These are 1034.8 ± 4.8; 829 ± 13 and 8121 ± 52 mg/kg DW, respectively. To compare, orange and lemon juice contains 100 ± 12.2 and 69.2 ± 4.02; 90 ± 7.65 and 63.5 ± 1.32; 1620 ± 156 and 1257 ± 73.1 mg/L respectively [40]. The last element with the highest content was sodium (564.3 ± 6.2 mg/kg DW). Sodium is responsible for maintaining the acid-base balance, the disruption of which can result in dangerous acidosis, which can cause osteoporosis, among other diseases. As an electrolyte, it is responsible for maintaining the internal water balance, as are potassium, magnesium, and calcium.

Micronutrients are trace elements that every living organism needs to function properly. For humans, the micronutrient requirement is less than 100 mg per day. Micronutrients can have different effects on the body. Iron, a component of hemoglobin responsible for transporting oxygen to the tissues in the body [41] was determined, in this study, in amounts of 16.578 ± 0.029 mg/kg DW. This is the comparable value as determined in the lemon juice samples from Argentina (15.6 ± 1.18 mg/kg). Similar results were also obtained to determine molybdenum—at 0.20 mg/kg [42]. In turn, zinc, which is involved in DNA and RNA synthesis and protein and insulin synthesis, was determined at 3.750 ± 0.077 mg/kg DW in the present study. Several essential micronutrients, including V, Ni, Cr, and Co, were below the defined LOD limits. The high content of microelements is an essential aspect of producing dietary supplements, drugs, animal food or cosmetics from plant raw materials. Therefore, kaffir lime juice may be applied in the food, agriculture, pharmaceutical, and cosmetic industries.

Most of the undesirable elements (Cd, Pb, Pt) in the diet were not determined (<LOD) or were determined at very low levels (Sr, Ba). An example of elements being determined is mercury (2.995 ± 0.013 mg/kg DW). Interestingly, the fourth highest elemental concentration determined was Al (100.87 ± 0.81 mg/kg DW). Aluminum does not show exaggerated toxic effects on the human body. Nevertheless, its excess is not welcome and can cause some damage to health. However, considering that the given values refer to the juice’s dry weight (moisture of citrus juices is almost 90% [43]) and the provisional tolerable weekly intake value is 1 mg·kg^−1^ body weight/week [44], the aluminium contained in the samples does not pose a health risk human.

Although micro- and macronutrients are not isolated from citrus fruits, according to Barros et al., citrus fruits are promising sources of mineral elements [45]. Citrus fruits are good sources of minerals, including potassium, calcium and magnesium, compared to other fruits [46]. These compounds are responsible for the body’s water and electrolyte balance. Therefore, the addition of kaffir lime juice may have potential use as an addition to rehydration drinks. Compared to other fruits, cherries, considered to be a good source of potassium, contain 2900 ± 50 mg/kg DW. The content of micro- and macroelements is also very important in the potential use of Kaffir lime juice as an additive to cosmetics. In cosmetic preparations, iron supports skin regeneration. In turn, zinc is a strong antioxidant, significantly delaying aging. It positively affects collagen metabolism and is responsible for the growth of hair, nails, and tissue regeneration.

### 2.4. Fluorescence Properties of Kaffir Lime Juice

**Figure 5:**
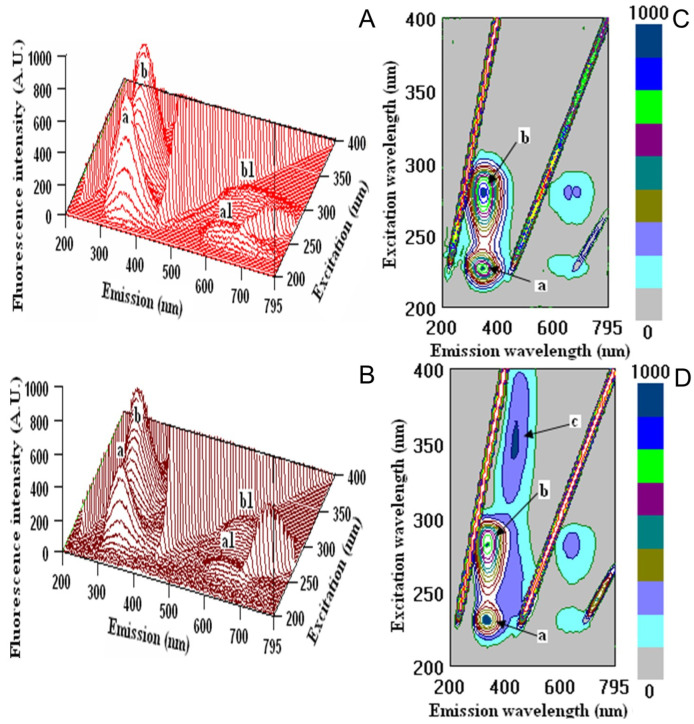
Fluorometric measurements in three-dimensional fluorescence analysis (3D–FL) of: (**A**)—HSA and kaffir lime juice after interaction (**B**) and cross images of: (**C**)—HSA (**D**)—HSA+ kaffir lime juice. The locations of peaks a, b, c, a1 and b1 are shown in the figure and in Table 8 (for interpretation of the references to colour in this figure legend, the reader is referred to the web version of this article).

Terpenes are nearly present in all-natural products. Human Serum Albumin (HSA) forms stable complexes with several substances. HSA binding can influence the bioactivity of terpenes, and albumin can also be considered and applied as a relatively cheap affinity protein. Therefore, we examined the potential interactions of the main terpene with HSA employing fluorescence spectroscopy. The flexibility of albumin’s structure is due to its organization into three domains, I, II and III. Each is subdivided into two subdomains, A and B. Intramolecular disulfide bonds ensure rigidity within each protein subdomain, but allow significant modifications in the shape and size of albumin in response to pH changes or other biophysical influences [47]. Some recent reports showed the interaction of terpenes with HSA, forming complexes, as with many compounds and drugs [48]. The binding of drugs to HSA determines their distribution through systemic circulation and its pharmacological effects on the organism [49], while terpenes are considered drugs and pharmaceutical agents [50]. Determination of terpenes’ binding to the main protein in blood human serum (HSA) is essential for human metabolism. The binding of terpenes to HSA under physiological conditions results from forming a complex. The fluorescence properties of lime extracts are presented in Figure 5.

The measurements were performed at the initial albumin (Alb) with λex/λem (nm/nm) = 227/349 and 279/353 with fluorescence intensity (FI, arbitral units) = 765.90 and 875.01 for peaks a and b, respectively. After interaction of HSA with kaffir lime juice, changes in λex/λem (nm/nm) = 231/334 with FI = 481.24 for peak a and λex/λem (nm/nm) = 282/339 with FI = 723.63 for peak b were detected. According to the decrease in the fluorescence intensities of peaks a and b, the binding properties were calculated (Table 8). The addition of kaffir lime to HSA showed increased binding of 54.5%. The relatively high percentage of binding properties of the bioactive compounds found in kaffir lime juice can be compared with the binding of HSA with different drugs. The interaction between paracetamol and HSA under physiological conditions has been investigated by fluorescence and showed similar results. Moreover, the protein-ligand docking study indicated that paracetamols (two paracetamols bind to HSA) bind to residues located in the subdomain IIIA [51].

The obtained results of kaffir lime juice can be as well compared with the interaction of HSA with one of the essential thiazole derivatives, 2-amino benzothiazole (2–ABT), which is widely used as a structural unit in the synthesis of antioxidants, anti-inflammatories, herbicides, antibiotics, and thermoplastic polymers. The interaction of 2–ABT with HSA under simulated physiological conditions by three-dimensional (3D) fluorescence showed high binding and fluorescence quenching spectra properties [52].

Recent reports showed wide use of the interaction of HSA with several mycotoxins. They proved that HSA binding could be applied as a relatively cheap affinity protein and used in vitro studies, as in our case with kaffir lime juice bioactive compounds [53].

As previously shown, HSA is widely used as an affinity protein. It is essential to show that not only polyphenols of natural products such as plants and fruits having high antioxidant activities form strong polyphenol-protein complexes in vitro and as well in vivo, based on the measurements of the binding properties of the substances under normal physiological conditions, as in the present study, but also terpenes. The levels of glycated proteins in the blood of diabetics are higher than that of non-diabetic subjects. The glycation of proteins is linked to the occurrence of diabetic complications and similar diseases, as it was shown in the reports that the glycation of HSA is believed to reduce the binding affinities for acidic drugs such as polyphenols and phenolic acids [54].

The binding affinities of kaffir lime juice are related to the bioactive properties of terpenes. The provided results suggest that terpenes in kaffir lime juice can be effectively transported and distributed in the blood after consumption. This creates new opportunities for the potential use of kaffir lime juice extracts in the pharmaceutical industry.

## 3. Materials and Methods

### 3.1. Samples

*Citrus Hystrix* fruits for analysis were transported from Thailand by the local distribution point in the Pomeranian Voivodship in December 2019. Fruits were provided to the laboratory in refrigeration conditions. From the information provided by the supplier, it appeared that the fruit was harvested in a similar degree of maturity and that the time since harvest was the same. Fruits were harvested manually. Samples were prepared and analyzed immediately after purchasing. Before the analysis, fruits were cleaned with the tap, rinsed with distilled water, dried using lint free paper towel and peeled manually. The juice was squeezed manually using a plastic juice squeezer to avoid oxidation processes and poured into a glass bottle. Immediately after squeezing, the juice was weighed into 20 mL SPME vials using plastic pipette tips. The vials were closed with caps with silicone Teflon membrane. Limes for the analysis were taken from 4 batches, each composed of 3 kg of fruits (70–80 pieces).

### 3.2. Reagents

Reagents for micro- and macroelements analysis were purchased from Merck (Darmstadt, Germany). Deionized water (Millipore—Milli-Q Water Purification System (Bedford, MA, USA)). was used throughout the study, and spectroscopically grade nitric acid (65%), supplied by Merck (Darmstadt, Germany), was used. Methanol, terpenes’ standards (ß-Pinene, Limonene, γ-Terpinene, α-Pinene, α-Terpineol, Camphene, α-Phellandrene, α-Terpinene, Terpinen-4-ol) were obtained from Sigma Aldrich (Schnelldorf, Germany). All chemicals, standards and reagents were of analytical grade.

### 3.3. Terpenes’ Analysis

#### 3.3.1. Optimization of Headspace Solid-Phase Microextraction (HS–SPME)

Carboxen/Polydimethylsiloxane/Divinylbenzene (CAR/PDMS/DVB) fibre with a thickness of 50/30 mm and a length of 2 cm (Sigma-Aldrich, St. Louis, MO, USA) was used for SPME. This fibre is recommended for flavour compounds by the manufacturer. It shows a strong extraction capacity for terpenic hydrocarbons, aldehydes, ketones and acids [55]. Before the extraction, the samples were kept at 40 °C for 5 min and agitated with a magnetic stirrer (750 rpm). Thermal desorption was set up to 250 °C for 5 min. Between each analysis, the fibre was cleaned at 250 °C for 2 min.

The influence of four independent factors on the yield of extraction of main terpenes from kaffir lime juice was evaluated using Fractional Factorial Design $2_{IV}^{4-1}$ using Minitab v17.1 Statistics Software (Minitab Inc. State College, PA, USA). A sum of peak areas of main terpenes (namely β-Pinene, Limonene, γ-Terpinene, α-Pinene, α-Terpineol, α-Phellandrene, camphene, α-Terpinene and Terpinen-4-ol) was considered as the response variable in the optimization of solid-phase microextraction—extraction yield. The type of fibre, extraction temperature, pH, sample volume, stirring speed, extraction time and ionic strength are related to SPME. Among these factors, the type of fibre, extraction temperature and extraction time are essential for volatile compound analysis [56]. The salt effect is also important, as it reduces the solubility of hydrophobic compounds and retains ionic strength [57]. According to the literature, terpenes have a high distribution coefficient between the coating and the sample; in this case, the amount of sample is a prominent factor [58]. As mentioned above, the fibre type was selected based on the manufacturer’s suggestions. For these reasons, the temperature and the time of extraction, the mass of salt added, and the mass of the sample were subjected to an optimization process (Table 9) and were selected for the FFD. The factors’ levels were selected based on previous research (to avoid overloading the detector at a given equipment sensitivity) and based on literature data [9]. Preliminary studies were performed to determine the required range of mass of the sample (X_1_), the mass of added salt (X_2_), extraction time (X_3_), and extraction temperature (X_4_). The whole experiment consisted of 11 runs (three replicates at the centres of the design). The experiments were performed in randomized order. The factors were denoted as −1 (low), 0 (central point) and +1 (high) to normalize the variables because of their different ranges and units (Table 9). Analysis of variance (ANOVA) was used to determine the adequacy of the factorial model. To select the optimal extraction conditions, Multi Response Prediction (MRP) was provided.

#### 3.3.2. Gas Chromatography

Gas chromatograph Agilent 7890A (Agilent Technologies, Palo Alto, CA, USA) equipped with a split/splitless injector and Pegasus 4D TOFMS (LECO Corp., St. Joseph, MI, USA) was used for analysis. The extraction step was made using an MPS autosampler (Gerstel Co., Mülheim, Germany). The nonpolar Equity-1 (Supelco, Bellefonte, PA, USA) column 30 m × 0.25 mm i.d. × 0.25 μm film thickness was utilized. The front inlet temperature was 200 °C, and the transfer line and ion source temperatures were set at 250 °C. The injector worked in split mode (ratio 1:100). The separation was achieved using the following temperature program for the oven: initial temperature 60 °C, ramped at 7.5 °C/min to 150 °C, then 15 °C/min to 250 °C and held for 2 min. The total time of analysis was 18 min. Helium (N6.0 class) was used as a carrier gas at a 1.0 mL/min flow rate. The detector voltage was 1716 V Mass spectra were collected from *m*/*z* 35–500 at ten spectra per second. The acquisition delay was 300 s. The internal standard method using borneol was chosen as the calibration method. The preliminary identification of the analytes was made by comparing the experimental spectra with those contained in the NIST 11 and Wiley libraries and by comparing the calculated retention indices. (RI) with literature values. RI values were calculated based on the C8–C20 n-alkanes analysis results. For each standard solution, the equation of the calibration curve and the coefficient of determination (R^2^) were determined. Validation parameters were also defined: limits of detection (LOD) and quantification (LOQ). The LOD and LOQ values were calculated based on the value of the standard deviation of the signal set (Sa) and the slope angle of the calibration curve (a).

### 3.4. Physicochemical Characteristics

Physicochemical parameters of the analysed juice included pH, titratable acidity, total soluble solids (°Brix), Brix/acid ratio, and vitamin C content. The pH was measured using a microcomputer pH meter (inoLab^®^ Multi 9310 IDS pH, WTW, Weilheim, 370 Germany). The total titratable acidity of the samples was established using the recommended method by the Association of Official Agricultural Chemists (AOAC) [59]. The titratable acidity of juice was measured by titrating with standardized 0.1 N NaOH until reaching pH 8.2. Citric acid was calculated based on the titrable acidity, assuming that the acidity in beverages is usually calculated as g/L citric acid [60]. Total soluble solids (°Brix) were determined using an OPTi Digital Handheld Refractometer (Bellingham + Stanley Ltd., London, UK). Vitamin C was determined iodometrically using the procedure of Trifunschi et al. [61]. All the tests were performed nine times, and the data obtained in the present investigation were subjected to statistical analysis of variance (ANOVA).

### 3.5. Inductively Coupled Plasma—Optical Emission Spectrometry Analysis

The first step in the sample preparation was lyophilization, carried out immediately after delivery of the samples. The lyophilized preparations were stored in sealed plastic bags at −20 °C. Concentrations of twenty trace elements (Na; K; Fe; Ca; Pt; Zn; Cd; Mg; Pb; Cu; Co; Ni; Mo; Al; Mn; Sr; Cr; Ba; V; Hg) were analyzed in all samples. The determination process of nineteen of these, except for Hg, was performed directly using a lyophilized sample and preceded by microwave-assisted mineralization. Multiwave Go microwave mineralizer (Anton Paar, Graz, Austria) equipped with a rotor and high-performance reaction vessels with pressure-activated-venting made of polytetrafluoroethylene-trifluoroacetic acid (PTFE-TFA) was used for the closed vessel microwave-assisted acid digestion of samples. About 0.5 g of lime samples were accurately weighted in the dried PTFE digestion vessels, and 8 mL of HNO_3_ was immediately added. The tightly closed vessels were placed in the microwave oven to digest the samples. The process lasted 50 min and consisted of five stages:

- Stage I: 10 min.—temperature rise to 100 °C,
- Stage II: 10 min. at 100 °C,
- Stage III: 10 min.—temperature rise to 180 °C,
- Stage IV: 10 min. at 180 °C,
- Stage V: 10 min.—temperature reduction to 60 °C.

After digestion, the contents of the reaction vessels were quantitatively transferred to 25 mL volumetric flasks. Each volumetric flask was then refilled with deionized water to a nominal volume. All prepared and blank samples were transferred to the polypropylene autosampler tubes for MP-OES analysis. The 4210 MP-OES supplied by Agilent has been used to determine most elements. Mercury/MA-3000 supplied by Nippon Instruments Corporation (NIC, Tokyo, Japan) was used to analyze mercury by cold vapour technique, and purified dry air was used as the carrier gas. The validation parameters of the analytical procedure are shown in Table 10.

### 3.6. Three-Dimensional Fluorescence Analysis (3D-FL)

The properties of bioactive substances in kaffir lime juice were determined by using three-dimensional (3D-FL) fluorescence (model FP-6500, Jasco spectrofluorometer, serial N261332, Tokyo, Japan) using the method of Kim et al. [62]. The 3D-FL was measured at emission wavelengths between 200 and 795 nm, and the initial excitation wavelength was 200 nm. All solutions for protein interaction were prepared in 0.05 mol/L Tris-HCl buffer (pH 7.4) containing 0.1 mol/L NaCl. The initial fluorescence intensities of albumin were measured before their interactions with the investigated samples. The changes in the fluorescence intensities were used to estimate the binding activities. The determination of the binding properties was done five times with an average.

### 3.7. Data Processing and Presentation

Data processing of chromatographic analysis was performed using a chromatographic peak deconvolution algorithm implemented in the software ChromaTOF^®^ (LECO Corp., version 4.44.0.0, St. Joseph, MI, USA). Microsoft^®^ Excel^®^ spreadsheet was used for data entry and calculations. Tables and charts were prepared using Microsoft Office Professional Plus 2016. GC chromatograms and 3D-FL images, and optimization plots were implemented from the software.

## Figures and Tables

**Table 1:** The Fractional Factorial Design for four factors with their observed responses.

DOE	Extraction Variables				Extraction Yield (Relative % to Maximum Yield)									
	X_1_	X_2_	X_3_	X_4_	ß-Pin	Lim	γ-Ter	α-Pin	α-Terpl	Camp	α-Phell	α-Terpin	Terpin	Sum
1	0	0	0	0	11.32	94.77	95.39	89.84	27.10	73.68	3.85	100.00	62.99	97.33
2	0	0	0	0	12.51	100.00	100.00	91.41	19.48	78.41	10.09	98.58	66.52	97.76
3	0	0	0	0	22.86	95.73	96.37	85.63	19.37	74.27	9.68	84.00	64.80	100.00
4	−1	−1	1	1	54.05	44.56	65.40	27.69	24.36	36.77	7.93	27.64	54.63	67.88
5	1	1	−1	−1	18.30	49.33	60.59	79.08	5.99	62.09	100.00	49.61	30.03	74.78
6	1	−1	1	−1	1.18	55.06	92.12	100.00	5.55	88.64	9.62	70.23	48.23	73.98
7	−1	−1	−1	−1	7.49	73.26	72.62	57.35	1.72	50.50	5.95	28.08	29.95	65.44
8	−1	1	−1	1	76.39	30.37	28.10	12.88	35.95	16.46	3.94	15.24	23.17	56.01
9	1	−1	−1	1	12.51	36.94	85.47	72.78	22.79	28.56	9.63	37.30	54.14	63.76
10	1	1	1	1	5.46	72.09	73.04	60.37	100.00	100.00	14.49	59.98	100.00	87.07
11	−1	1	1	−1	100.00	66.73	46.91	26.80	18.09	26.92	4.98	26.80	28.73	84.90

ß-Pin—ß-Pinene, Lim—Limonene, γ-Ter—γ-Terpinene, α-Pin—α-Pinene, α-Terpl—α-Terpineol, Camp—Camphene, α-Phell—α-Phellandrene, α-Terpin—α-Terpinene, Terpin—Terpinolene.

**Table 2:** Regression model for independent variables and their interactions.

Sum of Peak Areas of Main Terpenes in Kaffir Lime Juice			
	Mean Square	F-Value	*p*-Value
Model	2.92519 × 10^15^	141.91	0.007 *
Linear	1.60964 × 10^15^	78.09	0.013 *
X_1_	8.06023 × 10^14^	39.10	0.025 *
X_2_	1.25972 × 10^15^	61.11	0.016 *
X_3_	3.62883 × 10^15^	176.04	0.006 *
X_4_	7.44009 × 10^14^	36.09	0.027 *
2-way interactions	4.85738 × 10^14^	23.56	0.041 *
X_1_X_2_	3.42030 × 10^14^	16.59	0.055
X_1_X_3_	9.73402 × 10^13^	4.72	0.162
X_1_X_4_	1.01785 × 10^15^	49.38	0.020 *
R^2^	99.82%		
R^2^ (adjusted)	99.12%		

* Significant at *p* < 0.05.

**Table 3:** Coefficients of determination of a model of each chemical compound.

Chemical Compound	R^2^
ß-Pinene	98.13%
Limonene	99.74%
γ-Terpinene	99.77%
α-Pinene	99.80%
α-Terpineol	99.45%
Camphene	99.83%
α-Phellandrene	99.69%
α-Terpinene	98.30%
Terpinolene	99.88%

**Table 4:** The parameters of extraction of terpenes from kaffir lime juice selected during MRP.

Factors	Value	Unit
X_1_ sample mass	3.0	g
X_2_ mass of salt added	0.5	g
X_3_ extraction time	30	min
X_4_ extraction temperature	45	°C

**Table 5:** Selected validation parameters obtained based on the analysis of reference substances corresponding to the main monoterpenes identified in the volatile fraction of kaffir lime.

Terpene	RI_sample_	RI_lit._	Ref.	Cond.	a	b	R^2^	*n*	LOQ [µg/g]	LOD [µg/g]	Range		CV	Rec. [%]
											Min.	Max.		
Camphene	957.91	960.64	[14]	ZB1, 110 °C	0.0322	−0.1579	0.9956	7	25	8.3	25	252.5	3.07	125.88
Limonene	1031.96	1030.3	[14]	ZB1, 110 °C	0.0283	−0.3372	0.9954	7	19	6.8	19	424.8	2.85	108.92
ß-Pinene	981.04	981.73	[14]	ZB1, 110 °C	0.0335	0.0125	0.9949	7	39	13	39	1101	2.88	108.99
α-Phellandrene	1008.20	1006.7	[14]	ZB1, 110 °C	0.0324	−0.1394	0.9995	7	17	5.6	17	213.6	6.12	46.10
α-Pinene	942.93	941.85	[14]	ZB1, 110 °C	0.0284	−0.1267	0.9954	7	26	8.5	26	433.3	0.22	94.11
α-Terpinene	1021.58	1020	[15]	HP-101	0.0542	−0.5572	0.9935	7	31	10	31	211.9	9.17	93.98
α-Terpineol	1183.68	1179.4	[16]	DB1, 120 °C	0.0242	−0.116	0.9913	7	25	8.2	25	235.9	3.33	127.02
γ-Terpinene	1058.39	1055.8	[14]	ZB1, 110 °C	0.0542	−0.5572	0.9915	7	23	7.8	23	215.4	2.50	51.78
Terpinen-4-ol	1174.64	1170.8	[14]	ZB1, 120 °C	0.02	−0.2083	0.9944	7	28	9.2	28	471.21	1.97	105.45
Terpinolene	1085.55	1079.3	[14]	ZB1, 120 °C	0.0264	0.0175	0.9966	7	38	12.8	38	172.2	3.82	38.33

RI_sample_—Retention Index obtained during the analysis, RI_lit._—Retention Index taken from literature, Ref.—literature reference for RI_lit._, Cond.—conditions of analysis performed during measurements of Retention Indexes taken from literature, R^2^—Coefficient of determination, LOQ—limit of quantification, LOD—limit of detection, CV—coefficient of variation, Rec.—recovery.

**Table 7:** Physicochemical properties of kaffir lime juice.

Parameter	Value
Total acidity	7.474 ± 0.050
C_citric acid_	74.74 ± 0.50 g/kg
Brix	10.35 ± 0.70
pH	2.406 ± 0.086
°Brix/acidity ratio	1.385 ± 0.050
Vitamin C	22.31 ± 0.53 mg/100 mL

**Table 8:** Fluorescence and binding properties of kaffir lime juice.

Peaks	Indices	HSA	KL	Binding to HSA [%]	β-Pinene	Binding to HSA [%]
a	λ_ex_/λ_em_ (nm/nm)	227/349	231/334	-	228/349	-
	Int F_0_	765.90 ± 58.14	481.24 ± 42.11	37.2 ± 3.31	497.18 ± 45.71	35.09 ± 2.52
a1	λ_ex_/λ_em_ (nm/nm)	-	233/637	-	-	-
	Int F_0_	-	95.40 ± 8.13	-	-	-
b	λ_ex_/λ_em_ (nm/nm)	279/353	282/339	-	280/354	-
	Int F_0_	875.01 ± 79.11	723.63 ± 7.63	17.3 ± 1.50	760.21 ± 68.24	13.12 ± 1.21
b1	λ_ex_/λ_em_ (nm/nm)	-	283/644	-	-	-
	Int F_0_	-	129.78 ± 11.2	-	-	-
c	λ_ex_/λ_em_ (nm/nm)	-	347/436	-	-	-
	Int F_0_	-	169.44 ± 13.14	-	-	-

Abbreviations: F_o,_ fluorescence intensity in arbitral units (A. U.).

**Table 9:** Selected factors and their level of the Fractional Factor Design experiment.

Factors	−1	0	1	Unit
X_1_ mass of the sample	1	3	5	g
X_2_ mass of salt added	0	0.5	1	g
X_3_ extraction time	10	30	50	min
X_4_ extraction temperature	30	45	60	°C

**Table 10:** Validation parameters of the procedure for determining selected elements in kaffir lime juice samples.

Analyte	Wavelength [nm]	LOD [mg/kg]	LOQ [mg/kg]	Linearity				
				Calibration Range [mg/kg]		Number of Meas. Points	Number of Repetitions	R^2^
				min.	max			
Na	568.263	1.1	3.3	10	200	5	4	0.9998
K	766.491	0.16	0.48	2.5	20	4	4	0.9997
Fe	371.993	0.33	1.0	1.0	100	8	4	0.9997
Ca	430.253	2.0	6.0	10	250	6	4	0.9995
Pt	265.945	0.075	0.23	0.40	4.0	4	4	0.9994
Zn	213.857	0.19	0.58	0.58	10	9	4	0.9995
Cd	228.802	0.022	0.066	0.066	20	8	4	0.9998
Mg	279.553	0.40	1.2	1.2	40	6	4	0.9996
Pb	405.781	0.012	0.035	0.050	5.0	6	4	0.9999
Cu	327.395	0.026	0.077	0.30	20	6	4	0.9999
Co	345.351	0.012	0.035	0.050	1.0	5	4	0.9999
Ni	361.939	0.0070	0.021	0.10	20	7	4	0.9999
Mo	386.410	0.0060	0.018	0.018	20	9	4	0.9995
Al	396.152	0.088	0.26	1.0	100	8	4	0.9998
Mn	403.076	0.0064	0.019	0.019	1.0	5	4	0.9999
Sr	421.552	0.0045	0.013	0.013	40	6	4	1.0000
Cr	425.433	0.0027	0.0082	0.01	10	8	4	0.9999
Ba	493.408	0.21	0.63	0.63	3.0	4	4	0.9962
V	437.923	0.0057	0.017	0.017	20	9	4	0.9997
Hg	253.700	0.00096	0.0029	0.0029	0.10	10	3	0.9999

## Data Availability

The data presented in this study are available on request from the Corresponding author. The data are not publicly available due to privacy reasons.
